# Electrochemical metrics for corrosion resistant alloys

**DOI:** 10.1038/s41597-021-00840-y

**Published:** 2021-02-11

**Authors:** Clara Nyby, Xiaolei Guo, James E. Saal, Szu-Chia Chien, Angela Y. Gerard, Huibin Ke, Tianshu Li, Pin Lu, Christian Oberdorfer, Sarita Sahu, Sirui Li, Christopher D. Taylor, Wolfgang Windl, John R. Scully, Gerald S. Frankel

**Affiliations:** 1 Citrine Informatics, Redwood City, CA 94063 USA; 2 Department of Materials Science and Engineering, Ohio State University, Columbus, OH 43210 USA; 3 Department of Materials Science and Engineering, University of Virginia, Charlottesville, VA 22904 USA; 4 QuesTek Innovations LLC, Evanston, IL 60201 USA

**Keywords:** Metals and alloys, Theory and computation

## Abstract

Corrosion is an electrochemical phenomenon. It can occur via different modes of attack, each having its own mechanisms, and therefore there are multiple metrics for evaluating corrosion resistance. In corrosion resistant alloys (CRAs), the rate of localized corrosion can exceed that of uniform corrosion by orders of magnitude. Therefore, instead of uniform corrosion rate, more complex electrochemical parameters are required to capture the salient features of corrosion phenomena. Here, we collect a database with an emphasis on metrics related to localized corrosion. The six sections of the database include data on various metal alloys with measurements of (1) pitting potential, E_*pit*_, (2) repassivation potential, E_*rp*_, (3) crevice corrosion potential, E_*crev*_, (4) pitting temperature, T_*pit*_, (5) crevice corrosion temperature, T_*crev*_, and (6) corrosion potential, E_*corr*_, corrosion current density, i_*corr*_, passivation current density, i_*pass*_, and corrosion rate. The experimental data were collected from 85 publications and include Al- and Fe-based alloys, high entropy alloys (HEAs), and a Ni-Cr-Mo ternary system. This dataset could be used in the design of highly corrosion resistant alloys.

## Abstract (summary)

**Table:** 

Measurement(s)	pitting potential • repassivation potential • Crevice corrosion potential • pitting temperature • Crevice corrosion temperature • corrosion current density • passive current density • corrosion rate • corrosion potential
Technology Type(s)	digital curation
Factor Type(s)	alloy composition • temperature • pH • chloride ion concentration • alloy microstructure • alloy heat treatment

Machine-accessible metadata file describing the reported data: 10.6084/m9.figshare.13611623

## Background & Summary

Corrosion of metallic structures has a substantial impact on the economy. A 1998 study estimates the direct cost of corrosion to be 276 billion USD each year in the U.S. alone, or about three percent of the gross domestic product (GDP)0^1–3^. Therefore, the accurate assessment, control, and prediction of corrosion is of paramount importance. For metals that undergo uniform corrosion, the corrosion rate can often be obtained by mass loss measurements as a function of exposure time to a corrosive environment or by electrochemical measurements. However, for many corrosion resistant alloys (CRAs), either homogeneous solid solutions or multi-phase alloys, this assessment is more complicated because of the existence of a thin oxide on the alloy surface known as a passive film^4^. This film decreases the rate of uniform corrosion but can be susceptible to accelerated localized corrosion, including pitting corrosion, crevice corrosion, and stress corrosion cracking, all of which are associated with the local breakdown of passive films. These corrosion modes can result in unexpected, catastrophic failure, which should be avoided by all means.

**Fig. 1:**
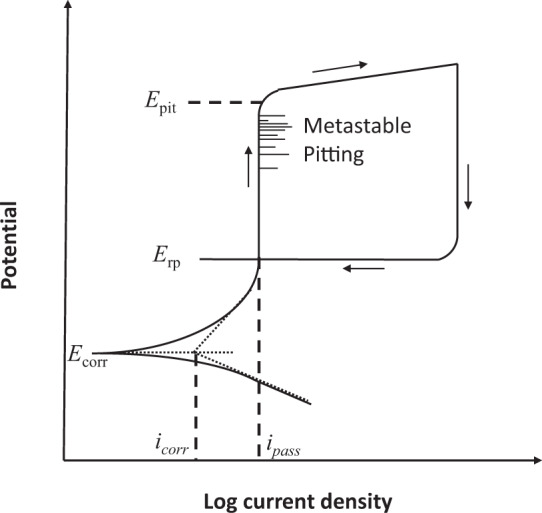
A typical cyclic potentiodynamic polarization curve, after Frankel, Journal of The Electrochemical Society, 1998, 145, 6^97^. Reproduced with permission.

Electrochemical approaches that are simple and fast have been widely used to assess the corrosion rate of CRAs, including linear polarization, potentiodynamic polarization, and electrochemical impedance spectroscopy (EIS)^5^. A number of electrochemical metrics derived from these techniques have been adopted to describe the corrosion resistance of alloys, such as corrosion current density (*i*_*corr*_), passive current density (*i*_*pass*_), corrosion potential (*E*_*corr*_), pitting potential (*E*_*pit*_), repassivation potential (*E*_*rp*_), crevice corrosion potential (*E*_*crev*_), pitting temperature (*T*_*pit*_, more commonly abbreviated CPT), and crevice corrosion temperature (*T*_*crev*_, more commonly abbreviated CCT). These metrics are defined in Table 1 and depend on the alloy composition, structure and defects, environmental factors including the temperature and chemical composition of the electrolyte, as well as physical factors such as the crevice former. A typical curve resulting from the cyclic potentiodynamic polarization of a passive metal is schematically illustrated in Fig. 1. Metals are electrochemically polarized from the active region toward the noble region by incrementally stepping the potential while the corresponding current density is recorded. The *E*_*corr*_ corresponds to the potential at which no external current flows. At this potential, the metal corrodes at a rate defined by *i*_*corr*_, which is the corrosion current density. This value is generally obtained by extrapolating the linear portion of the anodic and cathodic branches of the polarization curves to *E*_*corr*_. When the potential is scanned toward the more noble direction, a passive region exists where the current density is independent of the applied potential. The corresponding current density for this region represents the *i*_*pass*_. Although metastable pitting could occur in this region, no stable pit could form. When the applied potential is more noble than a specific range of values, the passive film breaks down and stable pits form, a process accompanied by a rapid increase of the current density. This characteristic potential is defined as *E*_*pit*_, which has been broadly used to determine the tendency of a given metal or alloy to breakdown. During cyclic polarization, the scan direction of the potential is reversed when the current density exceeds a predetermined value. Subsequently, the *E*_*rp*_ value can be frequently but not always achieved when the current density drops substantially, indicating the repassivation of the pit. This value is generally lower than *E*_*pit*_. Since stable pits can only form when the potential is more noble than *E*_*pit*_, and they can only propagate at a potential more noble than *E*_*rp*_, higher values of *E*_*pit*_ and *E*_*rp*_ suggest that the material is more resistant to pitting corrosion. Compared to the weight loss measurement, these electrochemical metrics possess a number of advantages: (1) They can be obtained much faster, particularly for CRAs that are intentionally designed with high corrosion resistance. (2) For parameters such as *E*_*pit*_, *E*_*rp*_, and *T*_*pit*_, the results can be reproducible with well-controlled experimental conditions. (3) They help to gain an understanding of the corrosion mechanism. Therefore, these electrochemical metrics have been extensively used in the metal corrosion community, and a vast resource of data is available in the literature.

**Table 1:** Definitions of various electrochemical metrics for metal corrosion.

	Electrochemical Metric	Definition
*i*_*corr*_	Corrosion current density	Current density on the metal surface describing the general corrosion rate of metals.
*i*_*pass*_	Passive current density	Current density in the passive region
*E*_*corr*_	Corrosion potential	Potential at which the anodic and cathodic reaction rates are equal so that the measured current changes sign at this potential when the potential is scanned as in potentiodynamic polarization
*E*_*pit*_	Pitting potential	Lowest potential at which stable pits form
*E*_*rp*_	Repassivation potential	Potential at which stably growing localized corrosion (pits or crevices) repassivates and stops growing
*E*_*crev*_	Crevice corrosion potential	Lowest potential at which stable crevice corrosion forms
*T*_*pit*_ (CPT)	Pitting temperature	Temperature above which stable pitting corrosion initiates
*T*_*crev*_ (CCT)	Crevice corrosion temperature	Temperature above which stable crevice corrosion initiates
	Corrosion rate	Sample thickness loss per unit time, sometimes converted from weight loss per unit time normalized by sample area

Given the hidden damage that localized corrosion introduces, with its frequent difficulty of detection and consequent potential for unforeseen catastrophic failure of high-value assets, designing materials with resistance to localized corrosion is of high priority. Various factors should be taken into account for such designs, including material composition, microstructure, tendency for passivity, and electrochemical activity of the surface. However, current approaches for the development of CRAs are primarily based on intuition, history of past successes, and trial and error. Although empirical models capable of predicting the corrosion resistance of a specific group of materials exist in the literature, they are strictly constrained to a very limited and particular range of compositional space. For instance, the pitting resistance equivalence number (PREN) was developed as a figure of merit by correlating the pitting corrosion resistance to alloy compositions for Fe-Cr-Ni alloys^6^. One relation characterizing the resistance of austenitic stainless steels to pitting corrosion is^7^:

1 $$PREN= \% Cr+3.3\times  \% Mo+19.4\times  \% N$$

where the coefficients simply describe the relative effects of Mo and N to that of Cr and the concentrations are in weight percent. Based on this equation, the pitting corrosion resistance of austenitic stainless steel can be primarily controlled by the amount of beneficial components, i.e. Cr, Mo, and N. While the PREN has been broadly used in the CRA industry, this equation cannot be extrapolated to compositions outside of those used to fit the equation, including high entropy alloys (HEAs) and aluminum alloys.

**Fig. 2:**
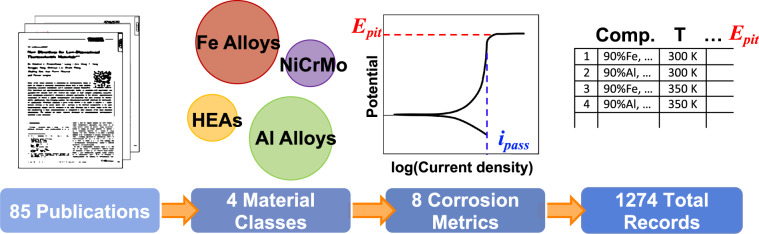
A schematic overview of the dataset. The data were collected from 85 publications, with materials which fall into 4 material classes. There are 6 datasets reporting a total of 8 different corrosion metrics, with 1274 total records.

In this study, we create a CRA database with electrochemical metrics that could be fed into machine learning (ML) based models to allow for exploration of the compositional space beyond what was used to create the current empirical models. To our knowledge, this is the first published database of this type. Existing large-scale databases only contain the uniform corrosion rate of certain types of alloys^8,9^. However, for CRAs, the uniform corrosion rate is not very meaningful because the dominant corrosion mechanism is localized corrosion as has been introduced above. The rate of the localized corrosion can be many orders of magnitude higher than that of uniform corrosion. Therefore, more complex electrochemical parameters, such as the pitting potential and repassivation potential or other metrics reported in this database, are required to capture these corrosion phenomena. A high level overview of the dataset is shown in Fig. 2. This database not only allows us to link the corrosion resistance of CRAs to various experimental parameters, including materials composition and environmental attributes (temperature, pH, and chloride ion concentration), but also enables the development of calculable matrices that could shed light on the fundamental physical processes that govern the corrosion performance. For instance, the corrosion resistance of metals could be correlated to the bonding strengths of metal-metal and metal-oxygen bonds^6,7^, chloride ion adsorption susceptibility^10,11^ and oxide enrichment and depletion factors^12^, which could be calculated through density functional theory or molecular dynamics approaches. ML models can also be integrated into a generic multi-physics modeling framework to bridge gaps where there is not yet mechanistic theory that enables the prediction of complex corrosion phenomena. Therefore, development of corrosion databases such as this may be crucial for the future of alloy design and optimization.

## Methods

The data were collected from experimental results reported across 85 literature sources^7,10,11,13–94^. Each composition was assigned a “Material class” based on the elements present and the source of the data. The Fe- and Al- based alloys are compositions that contain >35 wt. % Fe or Al, respectively. High entropy alloys are compositions that were reported as such in the source literature. The Ni-Cr-Mo ternary system contains alloys with all three elements present as well as some compositions containing just one or two of the three base elements. Alloy compositions which fall outside these bounds are classified as “Other”. The data for Fe-based alloys were collected from the references of a book^95^, while the data for the Ni-Cr-Mo ternary system of alloys were extracted from a doctoral dissertation^89^. The data for HEAs and Al alloys were collected from existing literature by searching the Corrosion journal, The Journal of Science and Technology, Science Direct, and Google Scholar. Some values were reported directly in each source, while the others were extracted from the figures using the WebPlotDigitizer software^96^. The database consists of six datasets containing different electrochemical metrics for CRAs including corrosion current density (*i*_*corr*_), passive current density (*i*_*pass*_), corrosion potential (*E*_*corr*_), pitting potential (*E*_*pit*_), repassivation potential (*E*_*rp*_), crevice corrosion potential (*E*_*crev*_), pitting temperature (*T*_*pit*_), and crevice corrosion temperature (*T*_*crev*_) (see Table 1 for definitions). Some sources provided average values of these descriptors, while others reported the maximum and minimum values. All of these values were recorded.

In addition to the electrochemical metrics, metadata related to details of the experiments were recorded. For every measurement reporting a corrosion potential, the experimental parameters include the alloy composition and environmental attributes of the corrosion experiment (temperature, test solution, pH, and chloride ion concentration). When provided, the alloy microstructure, alloy heat treatment, electrochemical testing method, and the electrochemical scanning rate were also recorded. For every measurement reporting a critical temperature as the corrosion metric, the experimental parameters reported include the alloy composition, test solution, and test method, with some measurements also including the scan rate of the temperature, test potential, alloy heat treatment, and alloy microstructure.

## Data Records

The data were collected from sources studying Fe-based alloys, HEAs, a Ni-Cr-Mo ternary system of alloys, and Al-based alloys. A description of the number of data records based on the electrochemical metric and material class is provided in Table 2. Beyond the reported electrochemical metrics and elemental composition, other fields that may be populated are:

- [Cl-]: Chloride ion concentration. It has been well established that the presence of chloride ions can break down the passive film of various CRAs and the extent of corrosion strongly depends on the concentration of these ions^97^. Therefore, it is crucial to include this parameter as an input for any model designed for the prediction of metal corrosion.
- Microstructures: Description of reported microstructures. In general, homogeneous solid solutions are more resistant to localized corrosion compared to multi-phase alloys. Localized corrosion can be initiated preferentially at heterogeneity on the surface, including dislocations and defects, secondary phase particles, inclusions, and grain boundaries, all of which are closely related to the microstructure of a given alloy. For example, the S-phase (Al_2_CuMg) particles existing on the surface of many Al-based alloys are more active than the matrix, so they are susceptible to localized breakdown. Similarly, pitting corrosion is usually initiated on MnS precipitates that exist in most stainless steels^97^.
- Oxide: Oxide layer composition, if present and reported. As described above, localized corrosion is a phenomena associated with the localized breakdown of the surface oxide, which strongly depends on the chemical composition of the oxide. The most widely used CRAs to date are primarily based on Fe-Cr and Ni-Cr, which rely on the formation of a Cr-rich surface layer that is highly resistant to aqueous corrosion. Therefore, the oxide composition, if known, will be beneficial for the understanding and prediction of localized corrosion.
- pH: pH of bulk test solution. Metal corrosion is influenced not only by the type of materials and potential, but also by the solution pH. The thermodynamic stability of a given metal or alloy in different pH environments can be found in the potential-pH diagram, also known as the Pourbaix diagram^98^. For example, under open circuit potential at room temperature, Fe can be strongly dissolved at acidic pH whereas passivisation occurs under basic conditions. Al corrodes slowly in a neutral environment but it is soluble in both acidic and alkaline environments.
- Scan rate of temperature: For temperature-dependent scans, this reports the scan rate of temperature in °C/min. For example, the determination of the *T*_*pit*_ relies on progressively increasing or decreasing the temperature at a specific step. This step sizes has been known to influence the measured *T*_*pit*_ values and their range of distribution^99^.
- Test Solution: Chemical composition of test solution. Corrosion is not an intrinsic property that only correlates to the material itself; it is also significantly influenced by the environment. The chemistry of the test solution is a critical environmental factor for metal corrosion and it directly determines whether localized corrosion will occur. In solutions without aggressive anions, predominantly chloride ions, localized corrosion does not occur, so additional care should be taken when interpreting the results obtained by electrochemical approaches. For instance, the rapid increase of current beyond the passive regime may be transpassive dissolution rather than pitting. Additionally, when the solution contains oxidizing agents, such as Fe^3+^, the likelihood of localized corrosion will increase. Similarly, if corrosion inhibitors are present in the test solution, the results should not be directly combined with those inputs collected in an inhibitor-free environment.
- Test temp.: Test temperature (°C). Corrosion is fundamentally governed by both thermodynamics and kinetics, so any changes in temperature will influence the rate of corrosion. An elevated temperature not only helps to overcome the energy barrier required for a given corrosion process to occur, but also accelerates the corrosion rate by simply enhancing the mass transport. In addition, many CRAs do not undergo localized corrosion until a critical temperature range is exceeded, which is usually expressed as the critical crevice temperature or critical pitting temperature. Recent studies showed that these parameters are statistically distributed rather than single-valued^99^. Due to the critical role temperature plays during corrosion, this input must be taken into account for the prediction of the corrosion rate for any metals.
- Test method: Type of electrochemical/chemical test performed, e.g. potentiodynamic, potentiostatic polarization, or immersion test. It is commonly observed that results acquired by different techniques can be slightly different, which should be considered if these inputs are combined.
- Scan rate (mV/s): The rate of potentiodynamic polarization scan in mV/s. During potentiodynamic polarization, the metals are polarized by incrementally increasing or decreasing the potential with a fixed step size, and the corresponding current density (i.e. corrosion rate) is measured. This electrochemical approach relies on the determination of surface reaction kinetics in a steady-state regime, otherwise the measured corrosion rate will deviate from the actual value, leading to inaccurate results that cannot be trusted. Therefore, the scan rate of potentiodynamic polarization must lie within a reasonable range for the results to be trusted, as has been reported elsewhere^100^.
- Heat treatment: Description of reported heat processing steps. Heat treatment predominately influences the structure of the alloys, so this input should be considered if provided.
- Reference: Literature reference number or DOI.
- Comment: If given, this may include information such as the name of a commercial alloy used in the test or other experimental details.

**Table 2:** The number of data records by electrochemical metric and material class. Here, “Fe” = Fe-based alloy, “Al” = Al-based alloy, “NiCrMo” = Ni-Cr-Mo ternary system alloy, “Other” = Other alloy.

Dataset	total	Fe	Al	HEA	NiCrMo	Other
*E*_*pit*_	810	523	118	27	92	50
*E*_*rp*_	189	45	41	9	92	2
*E*_*crev*_	31	31	0	0	0	0
*T*_*pit*_	117	116	0	0	0	1
*T*_*crev*_	70	35	0	0	0	35
*E*_*corr*_, *i*_*corr*_, and *i*_*pass*_	51	0	0	51	0	0

The database is available online on both the Citrination website^101^ as well as figshare^102^. On the Citrination website, the database can be downloaded as a collection of physical information files (PIFs)^103^, downloaded as an Excel spreadsheet, or used with the Citrination platform to build ML models. On the figshare website, the database can be downloaded as an Excel spreadsheet, in which the six datasets are present as individual tabs. The Fe-based alloys are presented in the spreadsheet without color highlighting and with the “Material class” column as “Fe Alloy”. The rest of the data include HEAs (orange color highlighting, “Material class”: “HEA”), Ni-Cr-Mo ternary system of alloys (green color, “Material class”: “NiCrMo Alloy”)), Al-based alloys (blue color, “Material class”: “Al Alloy”)), and a category containing materials which did not fall into any of the four material classes (purple color, “Material class”: “Other”)). An example section of the *E*_*rp*_ dataset (without color highlighting) is shown in Table 3, and the other datasets follow the same or a very similar format. The reference number corresponds to the references in the final tab of the dataset. This data can be straightforwardly incorporated for use in a ML framework.

**Table 3:** A section of the *E*_*rp*_ dataset. The full elemental composition includes the following elements: Fe, Cr, Ni, Mo, W, N, Nb, C, Si, Mn, Cu, P, S, Al, V, Ta, Re, Ce, Ti, Co, B, Mg, Y, and Gd.

No.	Composition (wt. %)										
	Fe	Cr	Ni	Mo	Avg E_*rp*_ (V_*SCE*_)	Temp (°C)	Test Solution	[Cl−] (M)	pH	Material class	Ref.
1	80.69	18.00	0.00	0.00	−504.0	60	3.5% NaCl	0.6	7	Fe Alloy	^48^
2	78.26	18.40	0.00	1.96	−478.9	60	3.5% NaCl	0.6	7	Fe Alloy	^48^
3	76.45	22.20	0.01	0.03	−403.5	60	3.5% NaCl	0.6	7	Fe Alloy	^48^
4	74.38	22.40	0.00	1.91	−465.9	60	3.5% NaCl	0.6	7	Fe Alloy	^48^
48	19.00	17.00	19.00	0.00	49.0	25	1 N H2SO4	1E-6	0	HEA	^18^
49	18.00	17.00	19.00	0.00	25.0	25	1 N H2SO4	1E-6	0	HEA	^18^
50	18.00	17.00	19.00	0.00	−156.0	25	1 N H2SO4	1E-6	0	HEA	^18^
51	18.00	16.00	18.00	0.00	−344.0	25	1 N H2SO4	1E-6	0	HEA	^18^
80	0.00	13.31	81.45	5.24	−33.8	45	0.5 M NaCl	0.5	7	NiCrMo	^44^
81	0.00	20.53	74.55	4.92	81.7	45	0.5 M NaCl	0.5	7	NiCrMo	^44^
82	0.00	16.95	74.64	8.41	−3.9	45	0.5 M NaCl	0.5	7	NiCrMo	^44^
83	0.00	28.67	65.94	5.38	90.68	45	0.5 M NaCl	0.5	7	NiCrMo	^44^
150	0.00	0.00	0.00	0.00	−806	25	0.6 M NaCl	0.6	7	Al Alloy	^56^
151	0.00	0.00	0.00	0.00	−754	25	0.6 M NaCl	0.6	7	Al Alloy	^56^
152	0.00	0.00	15.76	0.00	−358	25	0.6 M NaCl	0.6	7	Al Alloy	^56^
153	7.99	0.00	0.00	0.00	−304	25	0.6 M NaCl	0.6	7	Al Alloy	^56^

## Technical Validation

The data were collected and verified as accurate by a team of scientists familiar with all of the electrochemical metrics of corrosion reported here. Furthermore, additional screening was performed by examining outliers in various statistical plots of the datasets. Outliers were investigated and corrected if a transcription error or misinterpretation of the literature value was identified.

**Fig. 3:**
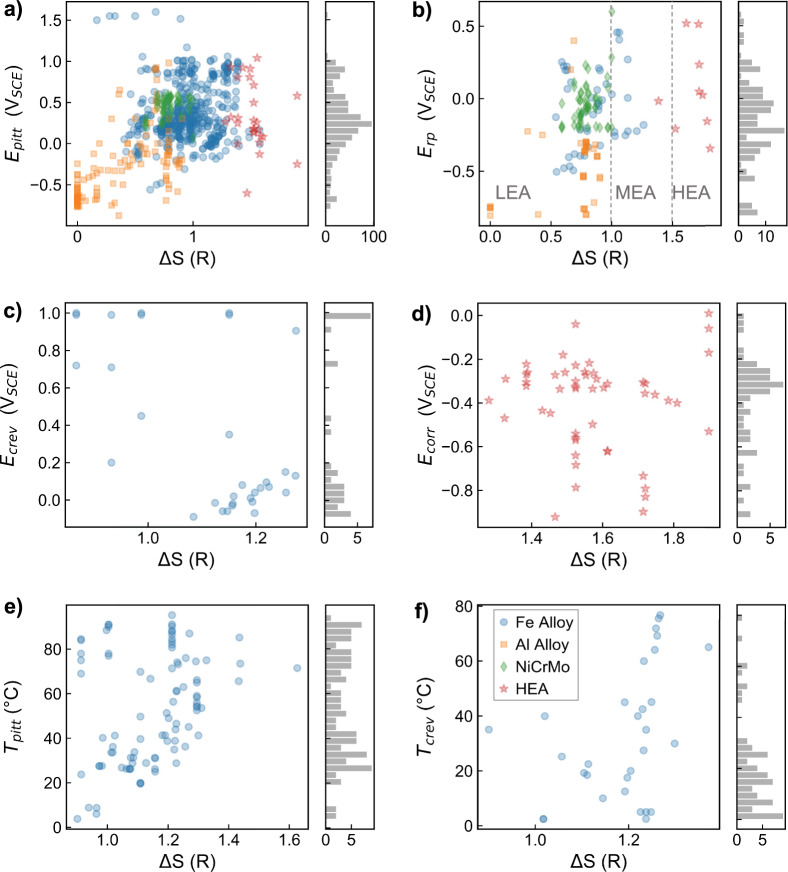
Configurational entropy of datasets. Here, we show the configurational entropy, Δ*S*, of the datasets reporting (**a**) *E*_*pit*_ in volts vs. saturated calomel electrode (V_*SCE*_), (**b**) *E*_*rp*_ in V_*SCE*_, (**c**) *E*_*crev*_ in V_*SCE*_, (**d**) *E*_*corr*_ in V_*SCE*_, (**e**) *T*_*pit*_ in degrees Celcius, and (**f**) *T*_*crev,max*_ (maximum of measured *T*_*crev*_ value) in degrees Celcius. The dashed lines in b) show the guidelines for classifying a low entropy alloy (LEA), medium entropy alloy (MEA) or high entropy alloy (HEA) based on the Δ*S* value. The histogram to the right of each panel shows the distribution in measured values for each corrosion metric.

This database contains compositions ranging from pure elements, multi-component high entropy alloys, and complex designed steels, with a total of 24 elements represented. To visualize the spread of the compositions in the data, Fig. 3 shows the configurational entropy, Δ*S*, for each of the six datasets. The Δ*S* of an alloy is given by^104^:

2 $$\Delta S=-\,R\mathop{\sum }\limits_{i=1}^{n}{x}_{i}ln{x}_{i}$$

where *n* is the number of elements, *x*_*i*_ the concentration of the *i*-th element and *R* is the universal gas constant. Note that this quantity represents the maximum possible configurational entropy for a given alloy composition, where this maximum value would be reached in the case of an ideal solid solution^105^. Different regimes of configurational entropy include low entropy alloys (LEA) having 0 < Δ*S* ≤ 1.5*R*, medium entropy alloys (MEA) in the range of 1 ≤ Δ*S* ≤ 1.5*R* and HEAs have Δ*S* > 1.5*R*^104^. We find that all of the classes of materials fall in a reasonable range, with the data points classified as HEAs in our database mostly above Δ*S* = 1.5*R*, the Al-based alloys falling in the LEA range (with some measurements on pure Al present at Δ*S* = 0), the Fe-based alloys spanning the LEA to MEA ranges, and the Ni-Cr-Mo ternary system of alloys falling in a cluster within the LEA range. These expected clusterings support the transcription accuracy of the elemental compositions collected in this database.

**Fig. 4:**
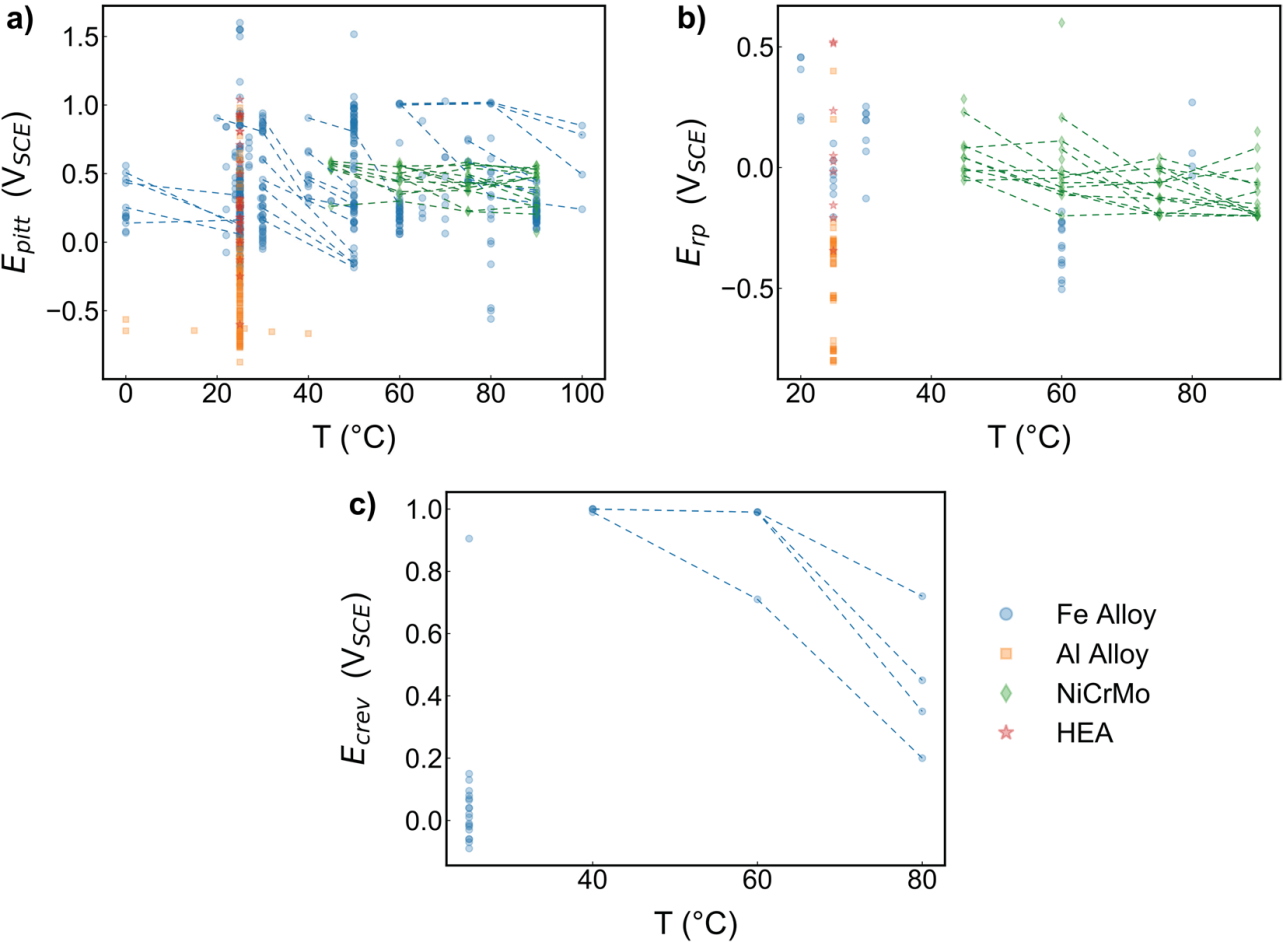
Temperature distribution of datasets. Here, we show the temperature distribution for (**a**) *E*_*pit*_ in V_*SCE*_, (**b**) *E*_*rp*_ in V_*SCE*_, (**c**) *E*_*crev*_ in V_*SCE*_. Dashed lines indicate measurements on the same material performed at varying temperatures.

In Fig. 4, we show the temperature dependence of the database, linking data from sources that included measurements at multiple temperatures. Here, we clearly see that the majority of the data were measured at room temperature. Indeed, all measurements for Al-based alloys and HEAs were measured at room temperature. Still, among the measurements with multiple temperatures for a given composition, we see a general decrease in *E*_*pit*_, *E*_*rp*_, and *E*_*crev*_ with increasing temperature, which follows the expected trend^97^, further supporting the validity of the dataset.

## Usage Notes

There are some cases in which the corrosion metric is given as a lower bound, rather than a real value. In particular, many of the entries in the Ni-Cr-Mo ternary dataset are reported as a lower bound for the *E*_*rp*_ potential because the corrosion resistance extended beyond the maximum potential of the electrochemical measurement. In order to use these data entries in a regression algorithm, these must be converted to a real number, with the method for conversion depending upon the intended application. Additionally, the content of some trace elements such as C, S, and P was often not provided by the original source. In those cases, the values for non-existing entries is reported as “NA”. Since the localized corrosion of several alloys depends on these trace elements, such as the role of sulfide in stainless steels, these fields may need to be replaced with estimated values. In some rare cases, the concentrations of some major elements such as Cr, Ni, and Mo were not provided by the original source and were therefore also reported as NA. These entries should be treated differently compared to the trace elements and should not be replaced with zeros. Detailed information on the specimens, when provided by the original source, was included in the “comments” field. This information includes materials grade, samples preparation details, surface finish, cleanliness, metallurgy methods, test area, and test location. It should be noted that the data are not uniformly distributed over material classes, elemental composition, or other features such as chloride concentration. Due to the nature of experimental corrosion data found in literature, it is impossible to achieve a perfectly balanced set of data with respect to all features. Therefore, the imbalance in the database should be taken into account when incorporating the data into statistical models.

Additional composition-dependent features could be generated for use in ML algorithms. For example, the Magpie featurization library could be used to generate features based on elemental and ionic properties, stoichiometry, and electronic structure^106,107^. Additionally, physics-based simulations could be performed to generate additional composition-dependent features. For example, the properties of alloys are influenced by the thermodynamic activity of each component, which can be calculated and used as inputs to the model. Furthermore, the severity of metal corrosion strongly depends on the bonding strengths of metal-metal and metal-oxygen bonds^108^. Specifically, alloying elements with strong metal-metal bonds, such as Mo, Nb, Ta, and W, do not corrode easily so they can act as dissolution moderators or blockers. Elements with weak metal-metal but high metal-oxygen bonds, such as Al, Ti, and Cr, can facilitate the formation of the surface passivation film. Therefore, the metal-metal and metal-oxygen bond strengths can be calculated^6,7^ as additional input features. Similarly, chloride ion adsorption susceptibility^10,11^ can be calculated because different alloying elements have different affinity toward the chloride ions, which are the dominant aggressive species that can break down the alloy passivity.

Care should be taken when combining input variables acquired from different environments. For example, pitting potential could vary with the solution aeration condition, particularly for alloys with a lower pitting potential than the corrosion potential. In naturally aerated solutions, the pitting potential of aluminum alloy 2024, for example, is close to its corrosion potential, *E*_*corr*−1_. However, in deaerated conditions, the corrosion potential strongly decreases (*E*_*corr*−2_) due to the absence of the oxygen reduction reaction, so the true pitting corrosion of this alloy can be revealed to be in the range of *E*_*corr*−2_ to *E*_*corr−*1_. Therefore, the solution aeration condition may be considered as a separate input variable. Additionally, some corrosion data were acquired in synthetic sea water, which has a much more complicated chemistry compared to other solution environments. For simplicity, a predictive model could use only pH and chloride ion concentration to approximate the solution chemistry. However, other species existing in this environment could also play a role during the corrosion measurement. Furthermore, some corrosion studies were performed in solutions saturated with oxygen or hydrogen, which may influence the redox environment and thus affect the measured electrochemical metrics.

## Data Availability

No custom code was developed for the generation or processing of this dataset.
